# Efficacy and safety of allogeneic platelet-rich plasma in chronic wound treatment: a meta-analysis of randomized controlled trials

**DOI:** 10.1038/s41598-024-75090-0

**Published:** 2024-10-24

**Authors:** Yalong Li, Xingtong Wang, Yucong Li, Dawei Li, Shijie Li, Chuanan Shen

**Affiliations:** Senior Department of Burns and Plastic Surgery, The Fourth Medical Center of PLA General, Beijing, China

**Keywords:** Allogeneic, Chronic refractory wounds, Platelet-rich plasma, Ulcers, Clinical effect, Meta-analysis, Skin diseases, Trauma

## Abstract

Allogeneic platelet-rich plasma (al-PRP) is gaining attention in clinical practice for treating chronic refractory wounds, though research results remain controversial. To assess the clinical efficacy of al-PRP for chronic refractory wounds. Databases including PubMed, Cochrane Library, Embase, CNKI, SinoMed, VIP, and WFPD were searched for randomized controlled trials comparing al-PRP with conventional treatments up to October 2023. Two researchers independently screened studies, extracted data, and assessed quality. Statistical analysis was conducted using RevMan 5.4, and potential publication bias was assessed and corrected using funnel plots and Egger’s test. Twelve studies with 717 cases were included. Meta-analysis showed al-PRP significantly improved outcomes compared to non-al-PRP treatments: increased healing rate (RR 2.72, 95% CI 1.77–4.19, *p* < 0.00001), shortened healing time (SMD − 1.03, 95% CI -1.31 to -0.75, *p* < 0.00001), improved efficacy rate (RR 1.19, 95% CI 1.10–1.28, *p* < 0.00001), increased wound shrinkage (MD 35.65%, 95% CI 21.65–49.64, *p* < 0.00001), and reduced hospital stays (MD -2.62, 95% CI -4.35 to -0.90, *p* = 0.003). Al-PRP is a feasible, effective, and safe biological therapy for chronic refractory wounds.

*Trial registration*: PROSPERO Identifier CRD42022374920.

## Introduction

Chronic refractory wounds, also known as recalcitrant or chronic wounds, lack a universally accepted definition globally. However, the generally accepted consensus is that a wound can be classified as chronic refractory if it fails to heal after one month of treatment or shows a lack of healing tendency. Such wounds are characterized by a healing rate of no more than 10-15% per week, or less than 50% contraction within a month^1^. Chronic refractory wounds encompass a wide range of types, including but not limited to chronic non-healing wounds due to infection, pressure ulcers, diabetic-related wounds, post-traumatic wounds, arteriovenous ulcers, and skin ulcers caused by radiation therapy. The incidence of these wounds is positively correlated with age and involves diverse and complex pathological mechanisms with a prolonged disease course^2^. In China, the proportion of surgical patients in hospitals with chronic refractory wounds ranges from 1.5 to 3.0%, with the most common types being post-traumatic wounds, pressure ulcers, and diabetic-related skin ulcers^3^. Chronic refractory wounds significantly extend hospital stays, increase treatment costs, and impose a substantial economic burden on families and society at large.

Research indicates that chronic refractory wounds from various causes can be effectively treated with subcutaneous injections of autologous platelet-rich plasma (PRP) and topical application of PRP gel, achieving favorable outcomes. This demonstrates the potential safety and efficacy of autologous PRP in treating these wounds. Patients receiving this treatment have shown a significant reduction in wound size, with no notable side effects reported. Additionally, PRP’s role in inhibiting the release of inflammatory factors also contributes to reduced pain and inflammation^4,5^. However, the use of autologous PRP faces certain limitations. Many elderly patients with chronic wounds often suffer from malnutrition, hypoproteinemia, and moderate to severe anemia, making it challenging to collect sufficient whole blood to extract PRP. Furthermore, the quality of PRP may be compromised in patients with severe underlying conditions or poor health, affecting its biological functions.

Allogeneic Platelet-Rich Plasma (al-PRP) has emerged as a focal issue in both basic and clinical research as an alternative to autologous PRP. Several teams have conducted clinical studies using allogeneic PRP for chronic refractory wounds^6^. However, the results vary and there is a lack of large-scale clinical trials and comprehensive data analysis, which limits its widespread adoption and promotion. Additionally, although some literature reports that allogeneic PRP can improve tissue recovery in the short term, clinical outcomes have not shown statistically significant differences^7^.

Therefore, a meta-analysis was conducted on clinical randomized controlled trials (RCTs) comparing the treatment of chronic refractory wounds with and without the application of al-PRP. This analysis provided evidence-based medical insights drawn from clinical practice, evaluating the efficacy and safety of al-PRP in treating chronic refractory wounds and exploring new therapeutic approaches for their repair.

## Data and methods

### Literature retrieval strategy

The retrieval strategy involves decomposing the systematic evaluation questions into keywords or subject terms recognizable by the computer system, following the PICO (participants, interventions, comparisons, outcomes) principle. Logical operations are then used to form a retrieval query to search Chinese and English literature published from the inception of each database to October 2023. The English databases include the Cochrane Library, PubMed, and EMBASE, while the Chinese databases include CNKI, VIP, Wanfang Patent Database, and the China Biomedical Literature Service System (SinoMed). For gray literature, searches are conducted through dissertation and thesis databases of various countries, abstracts of academic conferences, clinical trial registration platforms, as well as manual retrieval and retrospective searches to minimize the risk of missing relevant studies. Retrieval strategy:

#1(Plasma) OR (Platelet-Rich) OR (platelet-rich Plasma) OR (allogeneic platelet-rich plasma) OR (allogeneic platelet-rich plasma) OR (allogeneic platelet) OR (allogeneic platelet-rich plasma gel) OR (allogeneic platelet gel);

#2((Chronic refractory wounds) OR (chronic nonhealing wounds) OR (Chronic refractory wound) OR (chronic nonhealing wound) OR (chronic wound) OR (chronic wounds) OR (Diabetic Foot) OR (diabetic ulcer) OR (venous ulcer) OR (pressure ulcer);

#3(#1AND#2).

### Inclusion and exclusion criteria

Inclusion criteria:

i. Subjects: Chronic refractory wounds include infection-induced wounds, pressure injuries (pressure ulcers), diabetes associated wounds, arterial ulcers, venous ulcers, and radioactive skin ulcers, regardless of age, sex, or race.
ii. Interventions: Interventions: The experimental group received al-PRP treatment, while the control group received standard routine treatment without al-PRP (including debridement, drainage, decompression, dressing coverage, etc.);
iii. Research type: RCT;
iv. Outcome indicators: The total efficacy rate, healing rate, wound healing time, wound shrinkage rate after treatment, and length of hospital stay.

Exclusion criteria:

i. Studies on the same research or repeatedly published;
ii. Studies with incomplete data, unable to extract or convert data;
iii. Studies on combined use of two or more therapeutic measures;
iv. Animal test, review, case report, case analysis and conference abstract and studies unrelated to this study.

### Literature screening and data extraction

To ensure data integrity and reliability, improve the efficiency of data extraction, and minimize the impact of subjective factors, two evaluators from different professional backgrounds independently conduct the inclusion and exclusion process, literature quality evaluation, and design of the data extraction table. A blind extraction method is used. The data extraction table includes the following information: researcher, year of publication, journal of publication, year of obtaining the research data, total sample size, sample size of the experimental group and the control group, average age of patients, location of chronic refractory wounds, type of study design, and outcome indicators.

### Bias evaluation included in the study

In this study, the Cochrane risk of bias assessment tool was used to evaluate the quality of the included studies. Each study was assessed for bias risk and categorized as “high risk of bias,” “uncertain risk of bias,” or “low risk of bias.” The assessment focused on six domains and seven items to determine the overall risk of bias for each included study.

### Statistical analysis

NoteExpress was used for study screening, and RevMan 5.4 was employed for the meta-analysis. The heterogeneity of the included studies was measured by the I^2^ statistic. If the heterogeneity test result was *P* > 0.10, it indicated that the studies were homogeneous, and a fixed effects model was used. If the heterogeneity test result was *P* ≤ 0.10, the causes of heterogeneity were analyzed, and a subgroup analysis was conducted to calculate the combined statistics. If heterogeneity persisted despite these adjustments, a random effects model was used. When describing the combined results of multiple similar studies, the RR and its 95% CI were used as the combined statistics if the analysis index was a binary variable. For continuous variables, the MD or the SMD and their 95% CI were used. The combined statistics were assessed using a Z test. If *P* ≤ 0.05, the combined results were statistically significant.

## Results

### Analysis of search results

**Fig. 1:**
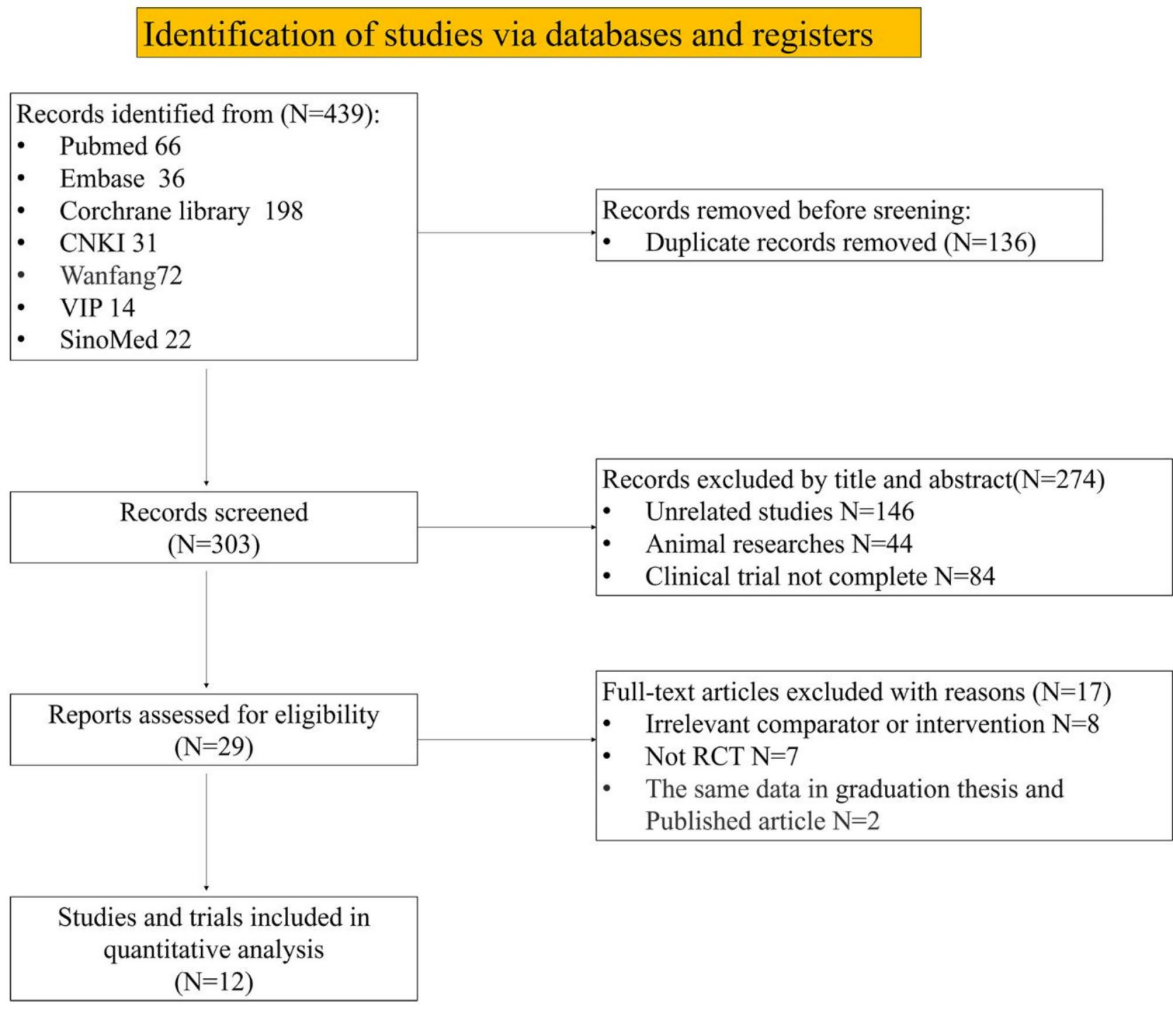
Flow chart of studies screening.

In accordance with the retrieval strategy, a total of 439 studies were retrieved. The studies were imported into NoteExpress, where duplicate studies were deleted. Studies that did not meet the inclusion criteria were then removed after reviewing the titles and abstracts. Ultimately, 12 studies were included for quantitative analysis (Fig. 1)^8–19^.

### Basic characteristics and quality evaluation of included studies

**Fig. 2:**
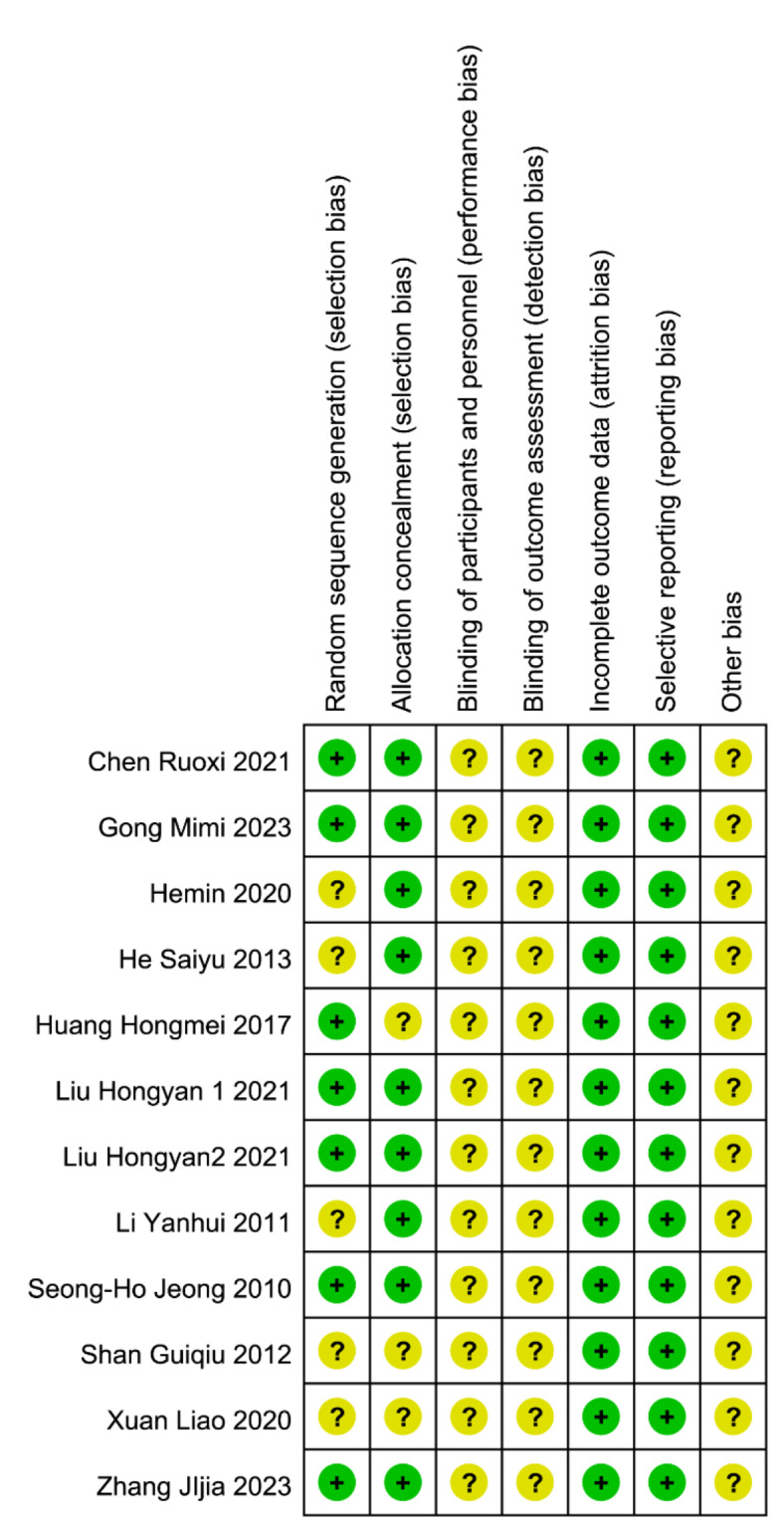
Summary of bias risk of the included studies (Note: “?” represents uncertain risk ,“+” represents low risk and “-” represents high risk).

**Fig. 3:**
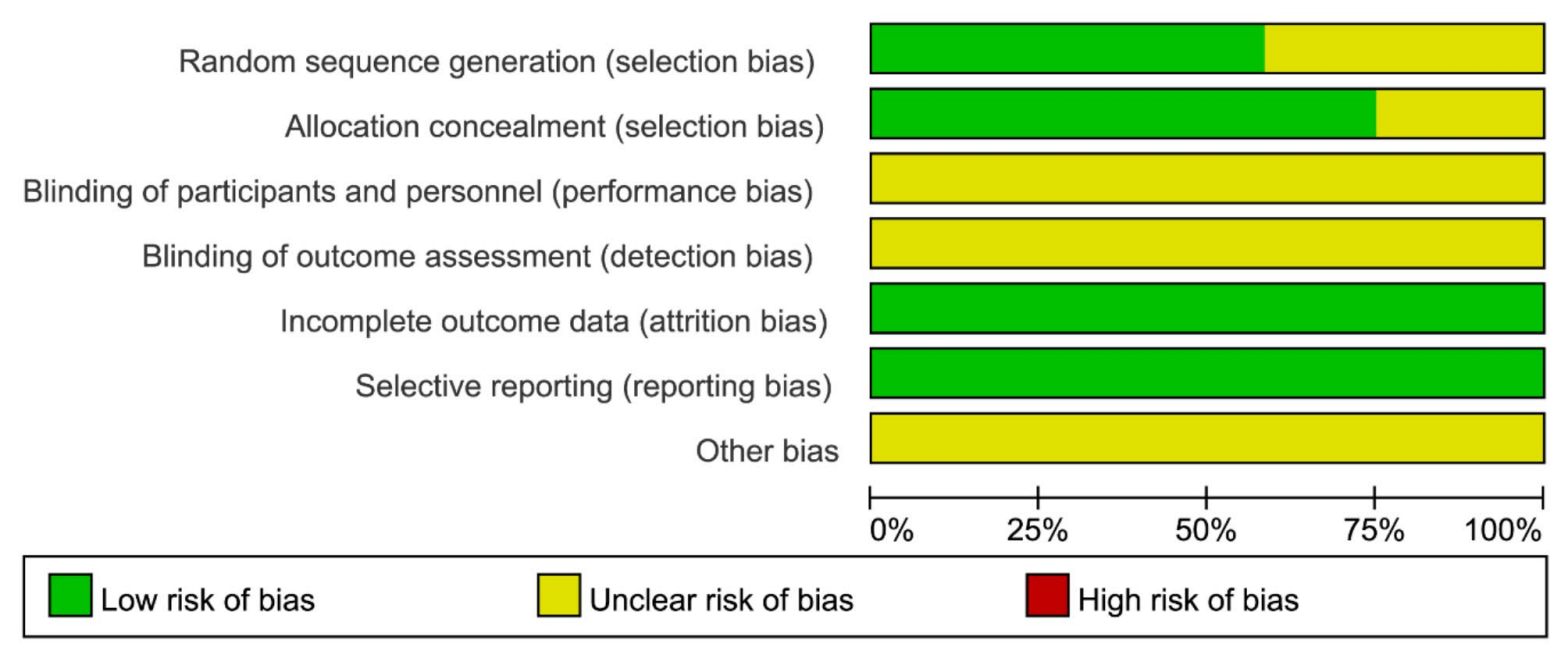
Bias risk of the included studies.

The 12 studies included in this analysis focused on subjects with chronic refractory wounds, with a total of 717 participants (358 in the experimental group and 359 in the control group). Table 1 details the basic characteristics of these studies, arranged in order of their publication date. Figures 2 and 3 present the results of the Cochrane risk of bias assessment for the included studies.

**Table 1:** Basic characteristics of the included studies.

Included studies and publication time	The time of recorded data	Total sample size (men/women)	Experimental group (men/women)	Control group (men/women)	Average age (Experimental /control)	Methods of randomization	Outcome indicator
Seong-HoJeong2010	2005.03-2006.12	100(53/47)	52(27/25)	48(26/22)	(64.5 ± 8.1/63.8 ± 6.4)	SAS version 9.1	①②④
Li Yanhui 2011	2010.01-2011.01	32(15/17)	16	16	68	not mentioned	①③④
Shan Guiqiu 2012	2010.01-2012.03	37	21	16	61	not mentioned	①③④
HE Saiyu 2013	2011.01-2011.12	60(34/26)	30	30	59 ± 2.6	not mentioned	①②③④
Huang Hongmei 2017	2016.05-2017.03	51(22/29)	26	25	59.5	A computer-generated table of random numbers	①②③
Hemin2020	2015.12-2018.08	50(36/14)	20(15/5)	30(21/9)	(66.3 ± 14.3/66.1 ± 12.1)	not mentioned	②
XuanLiao2020	2014.01-2018.01	60(39/21)	30(18/12)	30(21/9)	57 ± 10	not mentioned	④
Liu Hongyan 1 2021	2019.01-2020.06	86(48/38)	43(23/20)	43(25/18)	-(41.85 ± 5.91/42.19 ± 5.74)	Random number table method	②③⑤
Liu Hongyan 2 2021	2019.01-2020.05	78	39(25/14)	39(22/17)	-(43.02 ± 6.11/42.19 ± 6.08)	Random number table method	②⑤
Chen Ruoxi 2021	2014.01-2020.01	40(28/12)	20	20	72 ± 7	Random number table method	①②③
Gong Mimi 2023	2021.05-2022.10	40(24/16)	20	20	73	Random number table method	①②③④
Zhang Jijia2023	2020.01-2021.01	83(44/39)	41(21/20)	42(23/19)	(63.43 ± 7.32/62.32 ± 7.12)	Random number table method	②
Total		717	358	359			

① healing rate ② healing time ③ efficacy rate of treatment ④ wound shrinkage rate ⑤ hospital stay.

## Meta-analysis

### Healing rate of chronic refractory wounds

**Fig. 4:**
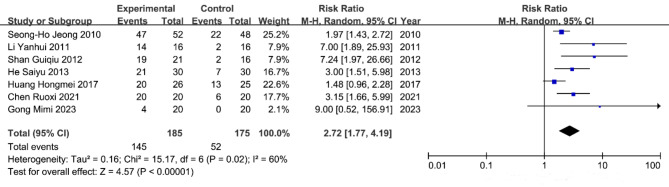
Healing rate of chronic refractory wounds.

A total of seven studies^8–12,17,18^ were included in the meta-analysis of the healing rate of chronic refractory wounds, encompassing 360 patients. The heterogeneity test results indicated heterogeneity among the study groups (*P* = 0.02, I^2^=60%); therefore, a random effects model was employed for the analysis. The results (Fig. 4) demonstrated that the healing rate in the al-PRP experimental group was significantly higher than that in the control group. The RR value was 2.72(95% CI 1.77–4.19), indicating a statistically difference between the two groups (*p* < 0.00001).

### Time required for wound healing

**Fig. 5:**
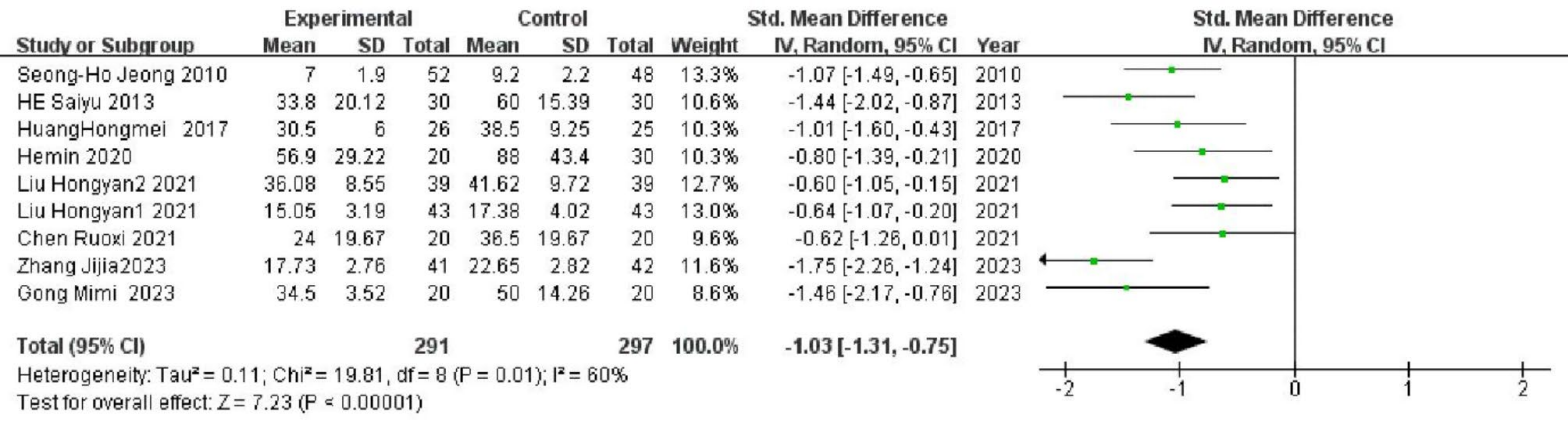
Healing time of chronic refractory wounds.

Nine studies^8,11–13,15–19^, involving a total of 588 patients, were included in the meta-analysis of healing time for chronic refractory wounds. The heterogeneity test indicated significant variability among the studies (*P* = 0.01, I^2^=60%), prompting the use of a random effects model. Due to differing units of healing time across the studies, the SMD was employed for comparison. The analysis (Fig. 5) revealed that the SMD for the healing time in the al-PRP experimental group compared to the control group was − 1.03, with a 95% CI of -1.31 to -0.75. This finding indicates that the experimental group experienced significantly shorter healing times than the control group, with a statistically significant difference (*p* < 0.00001).

### Efficacy rate of treatment

**Fig. 6:**
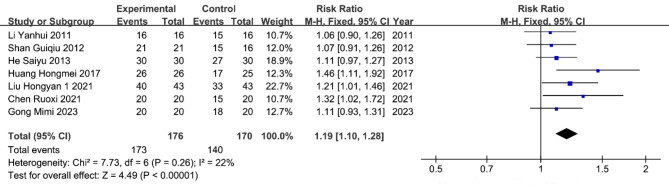
Efficacy rate of chronic refractory wounds.

Seven studies^9–12,15,17,18^, comprising a total of 346 patients, were included in the meta-analysis of the efficacy rate for treating chronic refractory wounds. The heterogeneity test indicated homogeneity among the study groups (*P* = 0.26, I^2^=22%), allowing for the use of a fixed effects model. The analysis results (Fig. 6) showed that the efficacy rate of al-PRP treatment in the experimental group was higher than in the control group. The RR value was 1.19(95% CI 1.10–1.28), indicating a statistically significant difference in the healing rates between the two groups (*p* < 0.00001).

### Wound shrinkage rate

**Fig. 7:**
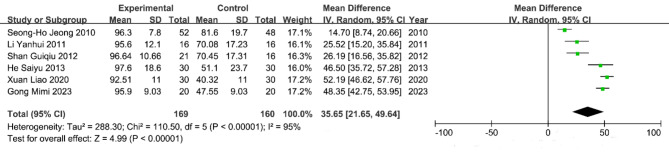
Wound shrinkage rate of chronic refractory wounds.

Six studies^8–12,14,18^, involving a total of 329 patients, were included in the meta-analysis of wound shrinkage rates for chronic refractory wounds. The heterogeneity test revealed significant variability among the studies (*P* < 0.00001, I^2^=95%), leading to the use of a random effects model for analysis. The results (Fig. 7) indicated that the wound shrinkage rate in the al-PRP experimental group was 35.65% (95% CI 21.65%, 49.64%), which was significantly higher than that in the control group (*p* < 0.00001).

### Hospital stay

**Fig. 8:**
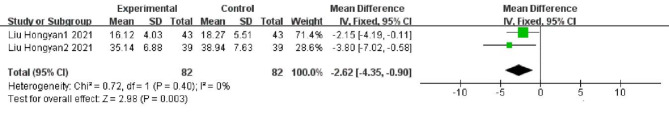
Hospital stay of patients with chronic refractory wounds.

Two studies^15,16^, with a combined total of 164 patients, were included in the meta-analysis of hospital stay duration for patients with chronic refractory wounds. The heterogeneity test indicated homogeneity between the study groups (*P* = 0.40, I^2^=0%), which justified the use of a fixed effects model for comparison. The analysis results (Fig. 8) revealed that the mean difference (MD) in hospital stay duration between the al-PRP experimental group and the control group was − 2.62 days, with a 95% CI ranging from − 4.35 to -0.90 days. Patients in the experimental group had significantly shorter hospital stays compared to the control group (*p* = 0.003).

### Sensitivity analysis

Sensitivity analysis can provide insight into the robustness of the combined effect estimate to a certain extent. In the analysis of the outcome indicator “wound shrinkage rate,” a one-by-one removal method was employed. It was observed that the heterogeneity originated from the study by Jeong^8^. Upon its removal, sensitivity analysis revealed a reduction in statistical heterogeneity between different studies (I^2^=89%, *P* < 0.01). The remaining four studies were analyzed using a random effects model, which still demonstrated a statistically significant difference in wound healing time between the control and experimental groups (*P* < 0.01). This finding underscores the robustness and reliability of the meta-analysis results. Similarly, sensitivity analysis was conducted for the other four effect indicators using the one-by-one removal method. The results consistently indicated statistically significant combined effect estimates, with the direction and significance of the original research results remaining unchanged. This further confirms the scientific validity and reliability of the meta-analysis results.

### Funnel plot analysis

**Fig. 9:**
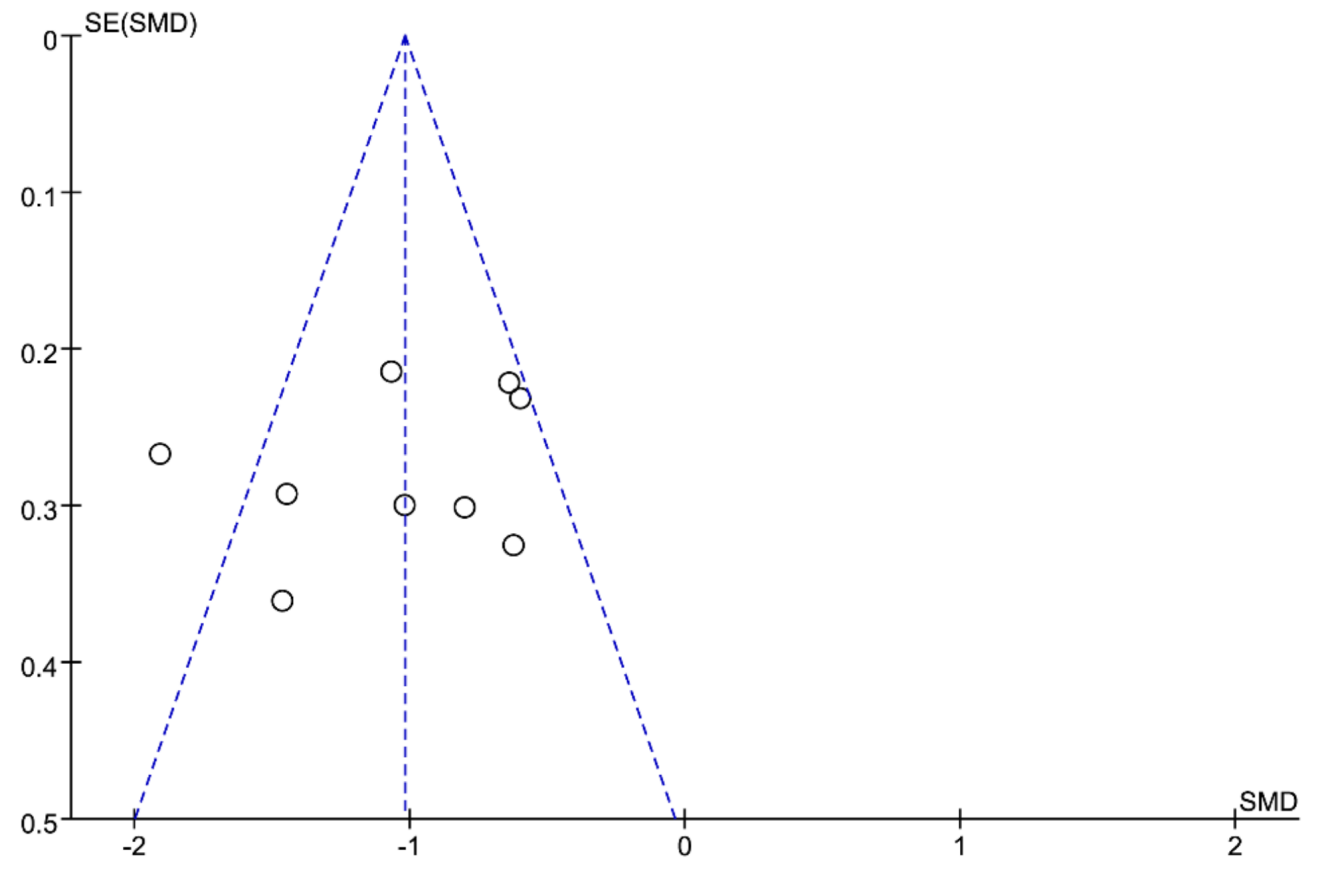
Funnel plot of publication bias of included studies.

**Fig. 10:**
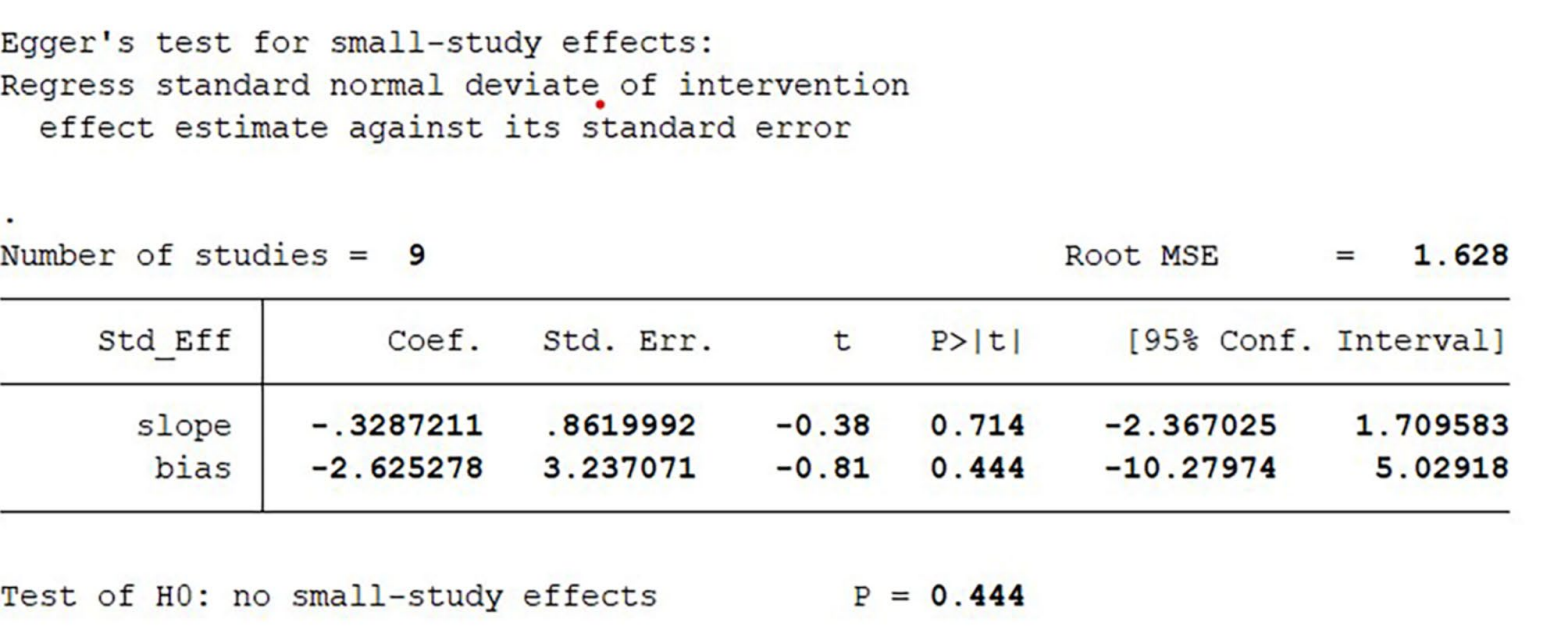
Egger’s test of included studies.

Funnel plot analysis is a method utilized to assess potential publication bias in research findings. In this study, “wound healing time” was selected as the indicator, and out of the 12 included studies, 9 used this measure for observation. The inclusion results (see Fig. 9) were randomly distributed throughout the funnel plot, appearing symmetrically across the middle and upper portions. We have conducted the Egger’s test using STATA 15 software, as shown in Fig. 10. The test results indicate a p-value of 0.44, suggesting that there is no significant publication bias among the included studies.

## Discussion

Skin wound healing is a sophisticated and tightly regulated biological process involving intricate interactions among various cell types, mediators, and signaling pathways^20^. The spontaneous wound healing process encompasses four stages, which may overlap or occur sequentially: (1) formation of a dynamic equilibrium of blood platelet embolism; (2) acute inflammatory stage; (3) cell migration and proliferation, encompassing extracellular matrix (ECM) formation and angiogenesis; (4) remodeling^21^. These stages are orchestrated by a myriad of cells, cytokines, and growth factors^22^.Chronic wounds present challenges in healing due to factors such as bacterial infection, retention of necrotic tissue, compromised blood circulation, inadequate growth factor availability, and disruption or abnormal apoptosis of the extracellular matrix^23^. These factors contribute to a significant increase in inflammatory cells and inflammatory mediators during the acute inflammatory phase, along with excessive secretion of matrix metalloproteinases (MMPs), which degrade the extracellular matrix^24^. Glycation of the ECM during cell migration, proliferation, and remodeling results in instability, reducing the content of type I collagen fibers, proteoglycans, and other components of the loose matrix. This inhibits cell proliferation and migration, as well as the formation of vascular granulation tissue, thereby disrupting the normal wound healing process and leading to prolonged and challenging wound healing^25^.

PRP is a plasma concentrate obtained from whole blood after centrifugation, comprising a variety of cell components, notably platelets in concentrations exceeding physiological levels^26^. Easily obtained through a straightforward centrifugation process, PRP represents a safe, simple, and cost-effective product^27^. While the molecular mechanisms by which platelet-rich plasma (PRP) promotes wound healing remain incompletely understood, several potential pathways have been proposed. First, PRP enhances angiogenesis, a critical process in the healing of chronic wounds. The formation of new capillaries improves blood supply, delivering oxygen, nutrients, and proteins, while facilitating waste removal^28^. Platelet α-granules, which are rich in growth factors such as platelet-derived growth factor (PDGF), fibroblast growth factor (FGF), and vascular endothelial growth factor (VEGF), play a crucial role in endothelial cell proliferation, prevention of apoptosis, increased vascular permeability, and angiogenesis^29^. Animal studies have shown that allogenic PRP significantly increases the number of regenerating blood vessels, thereby accelerating wound healing^30^. Secondly, PRP modulates the inflammatory response in wounds. By reducing pro-inflammatory cytokines such as interleukin-17 A (IL-17 A) and interleukin-1β (IL-1β), PRP alleviates inflammation, promotes vascular reconstruction, and supports rapid tissue repair^31,32^. It also regulates the AMP-activated protein kinase (AMPK) signaling pathway, influencing macrophage behavior by suppressing M1 macrophage differentiation and promoting M2 macrophage polarization^33^. M2 macrophages clear wound debris, induce CD4 T cell differentiation into regulatory T cells, and create a microenvironment conducive to tissue regeneration through the release of interleukin-10 (IL-10) and prostaglandin E2^34,35^. Thirdly, PRP stimulates skin cell proliferation, a key objective in restoring the skin’s barrier function. This involves fibroblast migration, extracellular matrix deposition, and keratinocyte proliferation^36^. PRP enhances epidermal stem cell (ESC) stemness and proliferative capacity by upregulating cytokeratin 19 (CK19), thus accelerating epithelialization^37^. Both in vitro and in vivo studies indicate that PRP promotes fibroblast proliferation, type I collagen production, and tissue remodeling^38,39^. Finally, PRP exhibits antibacterial activity, mediated by platelet-derived components such as antibacterial proteins and innate immune defense peptides^40^. PRP has shown significant efficacy against drug-resistant bacteria, including methicillin-resistant Staphylococcus aureus, vancomycin-resistant Enterococcus species, extended-spectrum β-lactamase-producing Klebsiella pneumoniae, and carbapenem-resistant Pseudomonas aeruginosa^41–44^.

Meta-analyses on the efficacy of autologous PRP in treating diabetic foot ulcers have demonstrated significant increases in healing rates, reduced healing times, and decreased amputation rates^45^. The 2020 China expert consensus on concentrated platelet products in wound repair also highlighted that concentrated platelet products are safe for chronic wounds, effectively promoting granulation tissue growth and re-epithelialization. These products surpass conventional wound treatments and can be used multiple times^37^. However, due to the lack of standardized methods for autologous PRP preparation, variations in preparation techniques and operator expertise result in differing platelet concentrations, leading to variable treatment outcomes. Additionally, the proportion of individuals over 65 years old is increasing year by year^46^. Many of these elderly individuals suffer from conditions such as diabetes, cardiovascular and cerebrovascular diseases, kidney diseases, and mobility impairments. They are often nutritionally deficient, presenting with moderate to severe anemia and hypoproteinemia, which can compromise platelet function and reduce platelet counts. Platelet counts in individuals over 70 years old are typically about 10% lower than in younger individuals^48^. Andia^49^also noted that the efficacy of autologous PRP injection treatment decreases with age in the context of osteoarthritis. Furthermore, each PRP preparation involves extracting tens to hundreds of milliliters of whole blood from the patient, potentially increasing their physical burden and exacerbating the underlying condition. Therefore, for patients unable to use autologous PRP due to these limitations, allogeneic PRP may be a viable alternative. Compared to autologous PRP, allogeneic PRP has broader sourcing options and easier collection methods. It can be derived from the whole blood of healthy individuals or blood banks, facilitating the extraction of an ample quantity of platelets with robust biological functionality through meticulous screening processes.

However, in vitro studies on allogenic platelet-rich plasma (PRP) have intriguingly suggested that the immune response elicited by allogenic PRP in a therapeutic context is generally minimal and potentially negligible^34^. Zhang^50^ conducted a pioneering in vivo study to assess the immunogenicity of allogenic PRP. In this study, allogenic PRP was intramuscularly injected into rabbits, resulting in only a slight, statistically insignificant increase in the number and proportion of CD4 + and CD8 + T cells in peripheral blood compared to pre-injection levels. Furthermore, histological examination of the injection site revealed no significant changes, leading to the conclusion that allogenic PRP exhibits low immunogenicity. Several factors may contribute to the low immunogenicity observed with allogenic PRP: (1) Limited Exposure to Host Antibodies: As a localized therapeutic agent intended to promote tissue healing, allogeneic PRP exhibits minimal interaction with host antibodies, thereby reducing the likelihood of triggering an immune response. (2) Altered Antigenic Structure: Platelet activation may modify their surface antigen structure and expression levels, potentially reducing their immunogenicity^51^. (3) Complete Degradation and Absorption: Allogeneic PRP is fully degraded and absorbed within a few weeks, eliminating the potential for a chronic immune response^52^. (4) Indirect Clinical Validation: The clinical use of allogeneic platelet transfusions further corroborates the low immunogenicity and high safety profile of allogeneic PRP. These findings suggest that while platelets possess immunogenic antigens, the risk of a significant immune response in the context of allogeneic PRP therapy is minimal, making it a potentially safe option for clinical applications in tissue healing. Furthermore, to ensure the safe use of allogeneic PRP, performing ABO and Rh blood typing, along with comprehensive pathogen screening—including HIV, hepatitis B, hepatitis C, and other bloodborne pathogens—is essential. This approach minimizes the risk of cross-infection and allergic reactions. Furthermore, maintaining strict sterility throughout blood collection, PRP processing, and application is critical to preventing microbial contamination.

This study found that the use of allogeneic PRP in the treatment of chronic refractory wounds is superior to conventional treatment methods without al-PRP. It significantly increases the healing rate, total efficacy rate, and wound shrinkage rate in patients with chronic refractory wounds, while also shortening the healing time. Therefore, it can effectively reduce the health risks and economic burden associated with chronic refractory wounds. Regarding the length of hospital stay, only two studies were included in the analysis, which makes the results less reliable. In all the included studies, neither the experimental group nor the control group reported significant local inflammation, allergies, or other adverse reactions. Despite the promising results of small-scale studies and our meta-analysis, there remains a significant gap in large-scale clinical trials assessing the efficacy and safety of PRP, particularly allogeneic PRP. The absence of such studies may be attributed to several factors, including the logistical complexity of organizing multi-center trials, ethical considerations surrounding the use of allogeneic materials, and the substantial financial investment required to conduct large-scale research. However, these challenges underscore the importance of pursuing large-scale, multi-center clinical trials in the future. Such studies are essential for providing robust evidence on the safety and effectiveness of allogeneic PRP, thereby supporting its broader adoption in clinical practice.

## Limitations of this study

There are still some limitations to this study. Firstly, the RCTs included in the meta-analysis were conducted in different patient populations and clinical settings. Although the detected heterogeneity is not significant, the risk of potential heterogeneity cannot be entirely ruled out. Secondly, since al-PRP treatment for chronic refractory wounds is a visible clinical procedure, blinding of doctors, nurses, and patients was not feasible. As a result, the included RCTs did not implement blinding, which might introduce unavoidable bias. Thirdly, variations in the preparation of PRP allografts, as well as differences in the methods and frequency of platelet concentration use, were not standardized across studies, contributing to heterogeneity. Additionally, due to the limited number of available studies, we included only 12 publications with a total of 717 patients, which may have limited our ability to capture the full range of variability and nuances in clinical practice. Lastly, most of the studies included were conducted in East Asia, which introduces geographical and healthcare system-related limitations.

## Conclusion

According to the results of our meta-analysis, the use of allogeneic PRP in the treatment of chronic refractory wounds is confirmed to be a feasible, effective, and safe biological therapy. However, heterogeneity exists among the analyzed trials. To enhance the reliability of research findings and provide a robust basis for clinical implementation, it is essential to conduct large-sample, multicenter, and well-designed randomized controlled trials across different regions and healthcare institutions.

## Data Availability

All data supporting the findings of this study are available within the paper. The data that support the fundings of this study are also available from the corresponding author upon reasonable request.
